# Diagnostic test accuracy of Xpert MTB/RIF for tuberculous pericarditis: a systematic review and meta-analysis

**DOI:** 10.12688/f1000research.22770.1

**Published:** 2020-07-22

**Authors:** Andrianto Andrianto, Ni Made Mertaniasih, Parama Gandi, Makhyan Jibril Al-Farabi, Yusuf Azmi, Michael Jonatan, Stevanus Immanuel Silahooij

**Affiliations:** 1 Faculty of Medicine, Universitas Airlangga, Surabaya, 60132, Indonesia; 2 Department of Cardiology and Vascular Medicine, Faculty of Medicine, Universitas Airlangga, Soetomo General Academic Hospital, Surabaya, 60286, Indonesia; 3 Department of Clinical Microbiology, Faculty of Medicine, Universitas Airlangga, Surabaya, 60132, Indonesia; 4 School of Management, University College London, London, UK; 5 Department of Internal Medicine, Faculty of Medicine, Universitas Airlangga, Soetomo General Academic Hospital, Surabaya, 60286, Indonesia

**Keywords:** Extrapulmonary TBC, Pericardial Effusion, PCR, Xpert MTB/RIF

## Abstract

**Introduction**: Xpert MTB/RIF is a rapid diagnostic instrument for pulmonary tuberculosis (TB). However, studies reported varied accuracy of Xpert MTB/RIF in detecting *Mycobacterium tuberculosis* in pericardial effusion.

**Methods**: We performed a systematic review of literature in PubMed, published up to February 1, 2020, according to PRISMA guidelines. We screened cross-sectional studies, observational cohort studies, and randomized control trials that evaluated the accuracy of Xpert MTB/RIF in diagnosing TB pericarditis. Papers with noninterpretable results of sensitivity and specificity, non-English articles, and unpublished studies were excluded. The primary outcomes were the sensitivity and specificity of Xpert MTB/RIF. We conducted a quality assessment using QUADAS-2 to evaluate the quality of the studies. A bivariate model pooled the overall sensitivity, specificity, positive likelihood ratios (PLRs), and negative likelihood ratios (NLRs) of included studies.

**Results**: In total, 581 subjects from nine studies were analyzed in this meta-analysis. Our pooled analysis showed that the overall sensitivity, specificity, PLRs and NLRs of included studies were 0.676 (95% CI: 0.580–0.759), 0.994 (95% CI: 0.919–1.000), 110.11 (95% CI: 7.65–1584.57) and 0.326 (95% CI: 0.246–0.433), respectively.

**Conclusions**: Xpert MTB/RIF had a robust specificity but unsatisfactory sensitivity in diagnosing TB pericarditis. These findings indicated that although positive Xpert MTB/RIF test results might be valuable in swiftly distinguishing the diagnosis of TB pericarditis, negative test results might not be able to rule out TB pericarditis.

**Registration**: PROSPERO CRD42020167480 28/04/2020

## Introduction

Pericarditis tuberculosis (TB) is the deadliest manifestation of extrapulmonary TB. TB is the primary cause of clinically signiﬁcant pericardial effusion in TB-endemic developing countries, responsible for as much as 90% of pericardial effusion in HIV-infected individuals and 50–70% in non-HIV-infected individuals ^1^. Mortality rates due to pericarditis TB rise six months after diagnosis, from 17% to 40% ^2^.

Early and accurate diagnostic measures are essential for tackling deadly extrapulmonary TB infections ^3^. Until 2018, Lowenstein-Jensen culture (LJ) was considered the gold standard for diagnosing TB pericarditis, since early non-invasive diagnostic procedures such as chest radiographs can only expose changes indicative of TB in 30% of cases, and echocardiography only presents a large frond-like pericardial effusion projection, which is nowhere near specific enough to indicate TB etiology ^4, 5^. However, LJ culture is time-consuming and complicated. Thus it is not recommended for use as a routine test ^6^. Other alternatives, such as histopathological examination, require invasive procedures and expertise that can only be found in a few medical centers. In addition, its sensitivity remains highly variable, ranging from 30 – 70% ^7^. Thus, histopathological examination is also not recommended for use as a routine test ^8^.

Currently, the Xpert MTB/RIF assay has FDA-approval as a diagnostic test of pulmonary TB. The Xpert MTB/RIF assay can detect members of the *Mycobacterium* complex and rifampicin resistance via nucleic acid amplification. This assay has been proven useful to diagnose pulmonary TB, with 89% sensitivity and 99% specificity ^7^. However, this method has not been established as a diagnostic test for extrapulmonary TB, such as pericarditis ^7^.

Several studies and meta-analyses have explored the benefits of using the Xpert MTB/RIF assay to diagnose extrapulmonary TB. However, to our knowledge, there is no meta-analysis of the use of the Xpert MTB/RIF assay in diagnosing pericarditis TB. Varied results, ranging from 59%–100% sensitivity and 72–100% specificity, have been found for the use of the Xpert MTB/RIF assay to diagnose pericarditis. Thus, this study intended to evaluate the accuracy of TB pericarditis diagnosis using a pericardial fluid sample for the Xpert MTB/RIF assay.

## Methods

### Study design

A meta-analysis was performed from February to April 2020 to assess the specificity and sensitivity of Xpert MTB/RIF for the diagnosis of pericarditis TB. This research was conducted according to the Preferred Reporting Items for Systematic Review and Meta-Analysis (PRISMA) statement and Cochrane Handbook for Systematic Reviews of Interventions. A checklist and review protocol adapted from PRISMA was used to guide the meta-analysis protocols in our present study (see *Extended data*). The systematic review protocol was registered with the International Prospective Register of Systematic Reviews (PROSPERO; identiﬁcation number CRD42020167480) on 28/04/2020.

### Eligibility criteria

Any document reporting diagnosis of pericarditis TBC using Xpert MTB/RIF assay based on primary data was considered. We included cross-sectional studies, observational cohort studies, and randomized control trials that evaluated the diagnostic accuracy of Xpert MTB/RIF for TB pericarditis. Exclusion criteria were (i) non-English articles; (ii) review, case-control studies, and case reports; (iii) studies that did not contain primary data in the published articles; and (iv) studies with noninterpretable results of sensitivity and specificity or had no samples that showed true positive (TP) and false positive (FP) results and/or true negative (TN) and false negative (FN) results. Duplicate data, commentaries, letters, correspondence articles, and editorials were excluded. Unpublished records such as conference papers, theses, and patents were not included in this meta-analysis.

### Search strategies and data extraction

Articles were systematically searched from inception to February 1, 2020, in PubMed databases. The search strategy conformed to medical subjects heading (MeSH) involving the use of the following keywords: ("tuberculous AND pericarditis") OR ("extrapulmonary OR pericardial OR pericardium") AND (tuberculosis) AND ("Xpert OR genexpert"). In our searching strategy, English language restrictions were applied. If the same data were used among studies, the more up-to date study with the larger sample size was preferred. The references cited by all the selected original research articles and reviews were searched for additional articles that might have been missed. Authors independently reviewed abstracts (Y.A, M.J, P.G, M.J.A, S.I.S) obtained from database searches and selected potentially eligible studies for full-text reviews. Data from included studies were independently extracted from eligible articles using a piloted data extraction form. The review authors extracted data independently on the following characteristics: name of the first author, year of publication, country of subjects, type of data collection, sampling method, patient characteristics and setting, the procedure for processing specimens, reference standards, and the results of the calculation of diagnostic test accuracy. All data were entered into a database manager, Microsoft Excel 2014. Disagreement between independent investigators was resolved by discussion and/or by consultation with the senior investigators (A, N.M.M).

### Quality assessment

To ensure quality and to avoid potential bias in each study, the quality of selected studies was controlled and assessed by two independent investigators (M.J.A, S.I.S). Included studies were appraised for their quality and bias using QUADAS-2. QUADAS-2 comprises four independent areas: subject preference, index test, source standard, and the flow and timing of patients through the study.

### Data analysis

We used the TP, FN, FP, and TN values reported in each included study to determine sensitivity and specificity. We used 95% confidence intervals (CIs) and generated the results graphically by plotting the data in forest plots. We plotted continuous diagnostic test results versus a gold standard in summary receiver operating characteristic (sROC) curves using Review Manager 5 (RevMan Cochrane, London, UK). The overall diagnostic sensitivity and specificity of included studies were pooled by a bivariate model using STATA 12 (STATA Corp, USA) with the "metandi" command.

## Results

### Summary of the included studies

**Figure 1.**
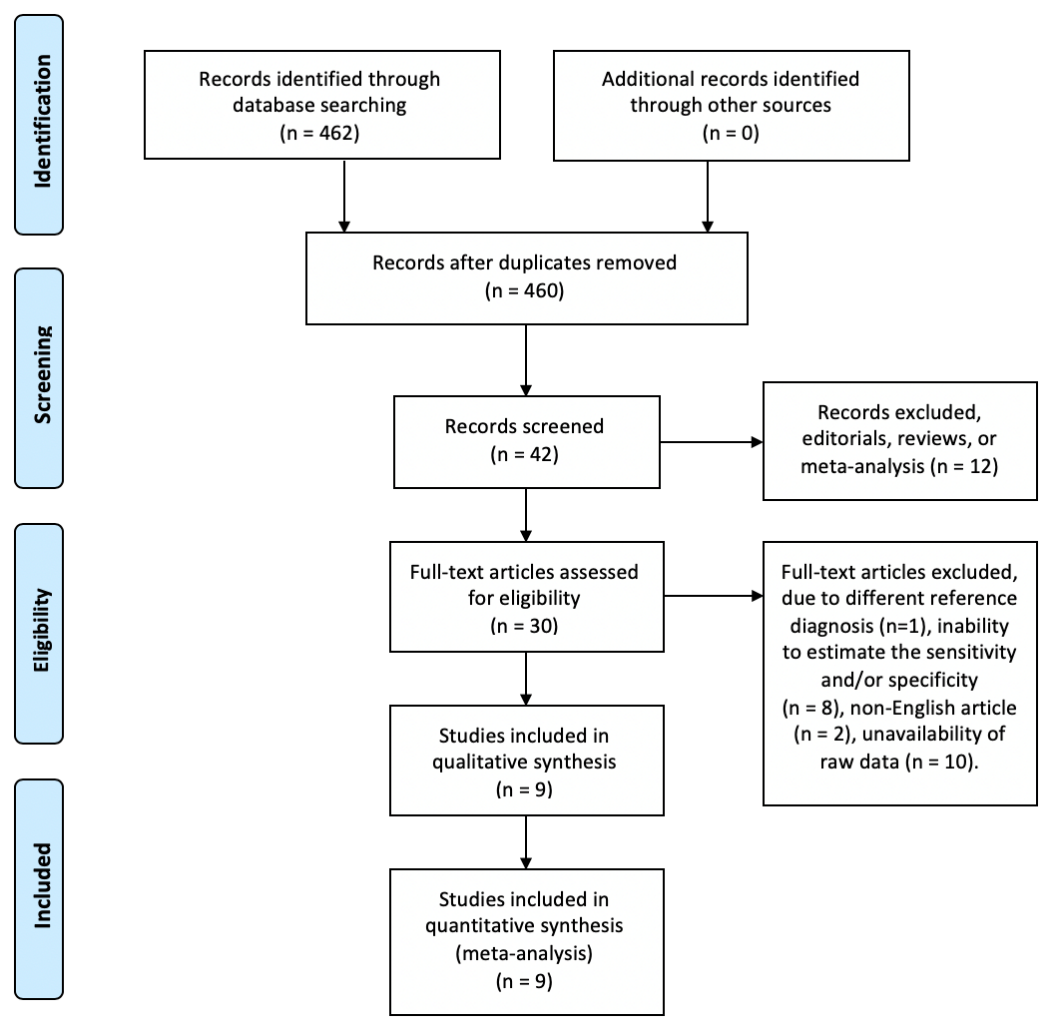
Flow diagram of the research collection.

After conducting literature screening, we included 30 studies for full-text article assessment. However, one study had different reference diagnoses, two studies were not in English, ten studies did not contain primary data in their published article, and eight studies had inadequate samples to calculate sensitivity and specificity; thus, they were excluded from our study. Therefore, in this meta-analysis, we included nine studies ^9– 17^. Figure 1 showed the flowchart of the research collection, while Table 1 provided a summary of the included studies. Of the nine studies included, three were carried out in Pakistan ^10, 13, 15^, two were conducted in South Africa ^12, 14^, and the rest were carried out in Turkey ^17^, Iran ^9^, China ^16^, and Spain ^11^. All studies used a prospective observational design. The number of pericardial effusion samples from each study ranged from three to 131, with a total sample size of 581 patients. All studies used a sample of patients with pericardial effusion, with an average prevalence of TB pericarditis of 28.4%. The MTB/RIF assay was applied to test the sensitivity and specificity in detecting Mycobacterium tuberculosis and resistance to rifampicin. The MTB/RIF assay ran nucleic acid amplification of the rpoB gene to detect M. tuberculosis, while resistance to rifampicin was assessed in an 81 bp region within the rpoB gene. As a diagnostic reference for MTB/RIF, a culture of pericardial fluid was used with solid and liquid media.

**Table 1.** Details of the included studies.

Authors	Country	Sample collection	Studied population	Total of the sample (TB/ non-TB)	PeE sample (TB/non-TB)	Reference for pericarditis TB
Allahyartorkaman, 2019 ^9^	Iran	2015–2018	Patients with suspected PTB and/or EPTB	220/1836	5/115	Culture/direct smear
Khan, 2018 ^10^	Pakistan	2013–2015	Patients with suspected EPTB	607/130	7/49	Culture
Moure, 2011 ^11^	Spain	1999–2011	Patients with suspected EPTB	108/41	1/2	Culture
Pandie, 2014 ^12^	South Africa	2009–2012	Patients with PeE	74/77	69/26	Culture
Saeed, 2017 ^13^	Pakistan	2014–2016	Patients with PeE and PuE	51/235	18/110	Culture
Theron, 2014 ^14^	South Africa	NR	Patients with suspected PTB and/or EPTB	46/85	46/85	Culture
Ullah, 2017 ^15^	Pakistan	2014–2015	Patients with suspected PTB and/or EPTB	88/178	4/16	Culture
Yu, 2017 ^16^	China	2016	Patients with PE	14/13	14/13	Culture
Zeka, 2011 ^17^	Turki	2010	Patients with suspected PTB and/or EPTB	89/340	1/5	Culture

EPTB, extrapulmonary tuberculosis; PTB, pulmonary tuberculosis; PeE, pericardial effusion; PuE, pleural effusion TB, tuberculosis; NR, not reported.

### Quality of the included studies

**Figure 2.**
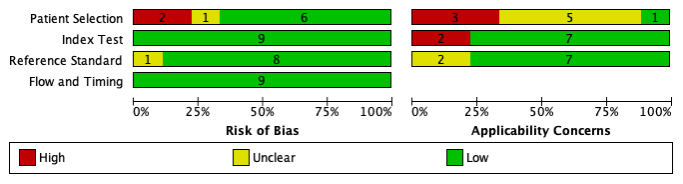
The author's review of each domain of risk of bias and applicability concerns are presented as a percentage of all the studies included.

**Figure 3.**
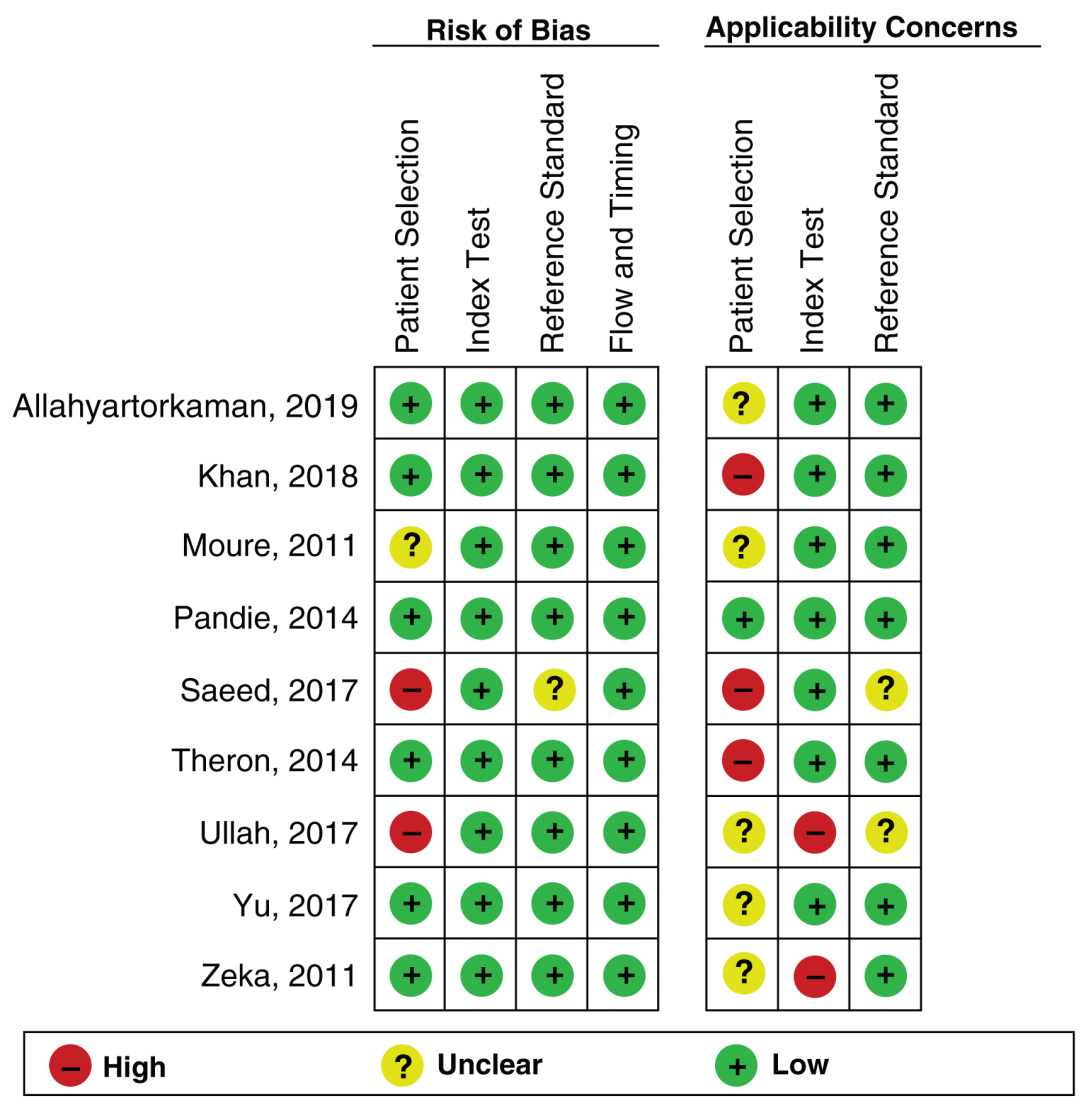
The author's review of each domain of risk of bias and applicability concerns for each included study.

We conducted a quality appraisal of included studies using QUADAS-2, and the results were shown in Figure 2 and Figure 3. QUADAS-2 examined four areas of the study, namely subject preference, index tests, source standards, and flow and timing. Each area, except flow and timing, consisted of two subdivisions, namely, risk of bias and applicability concerns related to patient characteristics and settings.

Regarding the risk of bias in the subject preference area, six studies ^9, 10, 12, 14, 16, 17^ were labeled as low risk, two studies ^13, 15^ were labeled as high risk, and one study ^11^ was labeled as an unclear risk because it did not explain the exclusion criteria clearly. The reason for the high risk of bias in this domain was due to inappropriate exclusion criteria. In the applicability concern section, we labeled one study as a low concern ^12^, three studies ^10, 13, 14^ as high concerns, and five studies ^9, 11, 15– 17^ as unclear concerns since the setting of patients was not mentioned. Studies in primary health settings were deemed low concern, whereas studies set exclusively in a tertiary care center were deemed high concern.

In the index test area, all studies included ^9– 17^ were labeled as having a low risk of bias. This was because Xpert MTB/RIF provided automatic results with a prespecified threshold test, and the user received the results on a printed sheet. In the applicability concern section, we grouped seven studies as low concerns ^9– 12, 14^ and two studies as high concerns ^15, 17^. To be categorized as a low concern, a minimum of 75% of the specimens in the study were required to have been processed according to WHO recommendations.

In the reference standard area, eight studies ^9– 12, 14– 17^ were labeled as having a low risk of bias, and one study ^13^ had an unclear risk of bias. To be categorized as a low risk of bias, the results of reference standards must be determined without the knowledge of the outcomes of index tests.

In the flow and timing area, all studies ^9– 17^ were considered to have a low risk of bias because all studies included more than 50% of eligible participants in the analysis.

### Diagnostic test accuracy of Xpert MTB/RIF for TB pericarditis

**Figure 4.**
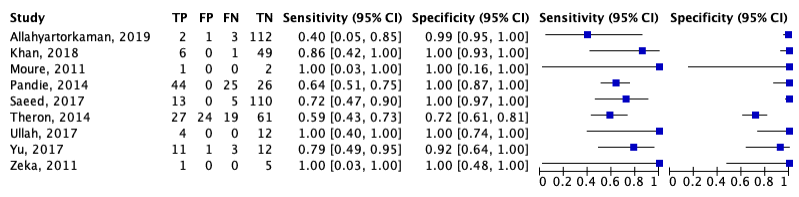
The approximated sensitivity and specificity of each of the included studies are plotted in forest plots. TP, true positive; FP, false positive; FN, false negative; TN, true negative.

**Figure 5.**
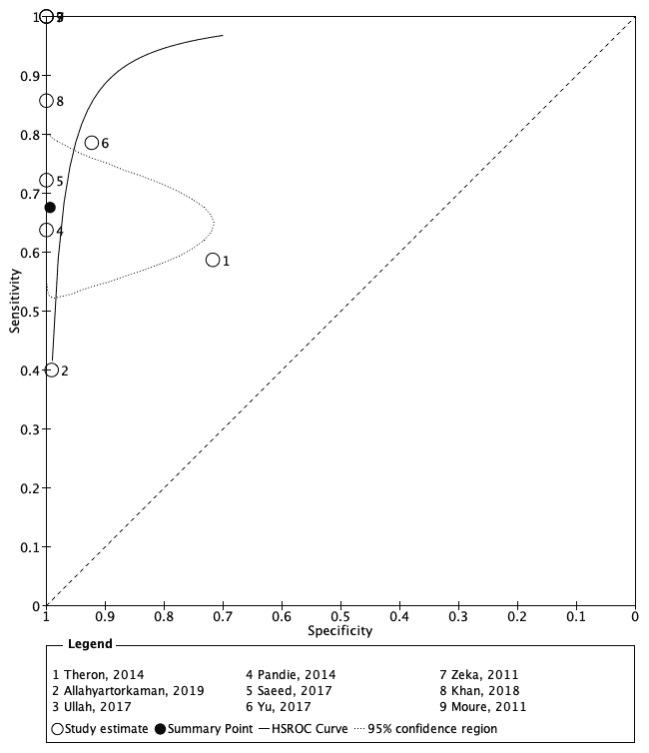
sROC for Xpert MTB/RIF. The black curve describes a pattern that considers culture as the perfect reference standard. The cleared circles outlined on several coordinates represent the estimated sensitivity and specificity of each of the included studies, and the black circle is a pooled estimate of sensitivity and specificity achieved from the bivariate model. sROC, summary receiver operating characteristic; HRSOC, hierarchal summary receiver operating characteristic.

Table 2 showed the significant findings from the included studies. In general, Xpert MTB/RIF had a high diagnostic accuracy in diagnosing TB pericarditis. The diagnostic test sensitivity and specificity forest plot of Xpert MTB/RIF for TB pericarditis were shown in Figure 4. Xpert MTB/RIF had an overall sensitivity of 0.676 (95% CI: 0.580–0.759), and the overall specificity was 0.994 (95% CI: 0.919–1.000). The PLR and NLR were 110.11 (95% CI: 7.65–1584.57) and 0.326 (95% CI: 0.246–0.433), respectively. Figure 5 showed a summary receiver operating characteristic (sROC) for Xpert MTB/RIF to illustrate continuous diagnostic test results versus the gold standard.

**Table 2.** Main findings of the included studies.

Authors	TP	FP	FN	TN	Sensitivity	Specificity
Allahyartorkaman, 2019 ^9^	2	1	3	112	0.40	0.99
Khan, 2018 ^10^	6	0	1	49	0.86	1.00
Moure, 2011 ^11^	1	0	0	2	1.00	1.00
Pandie, 2014 ^12^	44	0	25	26	0.64	1.00
Saeed, 2017 ^13^	13	0	5	110	0.72	1.00
Theron, 2014 ^14^	27	24	19	61	0.59	0.72
Ullah, 2017 ^15^	4	0	0	12	1.00	1.00
Yu, 2017 ^16^	11	1	3	12	0.79	0.92
Zeka, 2011 ^17^	1	0	0	5	1.00	1.00

TP, true positive; FP, false positive; FN, false negative; TN true negative.

## Discussion

To the best of our knowledge, this is the first meta-analysis that evaluates the diagnostic accuracy of TB pericarditis using Xpert MTB/RIF. Previously, six systematic reviews had assessed the diagnostic test accuracy of Xpert MTB/RIF for extrapulmonary TB. Chang *et al.*, 2012 ^18^ (eight studies) and Li Y *et al.*, 2017 ^19^ (26 studies), analyzed the diagnostic test accuracy of Xpert MTB/RIF for multiple forms of extrapulmonary TB combined. Denkinger *et al.*, 2014 ^7^ (18 studies), Maynard-Smith *et al.*, 2014 ^20^ (27 studies), Penz *et al.*, 2015 ^21^ (37 studies), Sehgal *et al.*, 2016 ^22^ (24 studies), and Kohli *et al.*, 2018 ^5^ (18 studies) analyzed the diagnostic test accuracy of Xpert MTB/RIF for specific forms of extrapulmonary TB. However, none of them specifically analyzed the diagnostic accuracy of Xpert MTB/RIF for TB pericarditis.

In this meta-analysis, the pooled specificity of Xpert MTB/RIF to diagnose pericarditis TB was extremely powerful for the majority of studies using Xpert MTB/RIF to diagnose extrapulmonary TB (0.994 (95% CI: 0.919–1.000), which highlights its effectiveness as a test to rule-in TB pericarditis diagnosis and help clinicians to decide on early TB treatment. This early TB treatment will be able to improve outcomes and reduce mortality for patients ^23^. Interestingly, this study showed better sensitivity and specificity than previous meta-analyses, which studied the use of Xpert MTB/RIF to diagnose various extrapulmonary TB ^5, 7, 18– 22^. This might be because a bivariate model pooled the overall sensitivity and specificity in this study. Thus, the validity of our results were higher than that of the prior meta-analyses. In addition, we limited the diagnosis to TB pericarditis rather than unspecified extrapulmonary TB.

The sensitivity of Xpert MTB/RIF for TB detection in pericardial fluid varied widely across the studies involved in this meta-analysis. Thus, the pooled sensitivity outcome was not very high (0.676 (95% CI: 0.580–0.759). The paucibacillary characteristics of the TB pericarditis might cause the reduced sensitivity of diagnosis with Xpert MTB/RIF using pericardial fluid ^24^. The detection limit of Xpert MTB/RIF is 131 CFU/ml; any unit lower than this cannot be detected, resulting in negative results ^25^. Hence, this may lead to a false-negative diagnosis of pericarditis TB. Other possible explanation are the variation in the sample size, sampling method, patient characteristics, and settings. In this meta-analysis, 45% of the included studies were conducted in areas with low prevalence. Patients in these areas may present with a paucibacillary disease earlier, which reduce the sensitivity of Xpert MTB/RIF ^26^. Limited sensitivity will hinder the usage of Xpert MTB/RIF for ruling out TB pericarditis, especially when samples are shown to be smear-negative. Meanwhile, LJ culture can detect an organism from as low as 10 CFU/ml to 100 CFU/ml ^27^, resulting in cell culture as the best reference standard for TB detection, especially when a very low number of organisms are involved. 

Though the interpretation is not straightforward, the sensitivity, specificity, and AUC under sROC are the fundamental aspects that demonstrate the accuracy of the diagnostic test. Nevertheless, in a clinical setting, NLR and PLR can be essential and exhibit the actual state. In order to identify and rule out target diseases, it is frequently accepted that a PLR above 10 and an NLR below 0.1 signify clinical significance ^28^. In this present study, the PLR and NLR values for Xpert MTB/RIF in the diagnosis of TB pericarditis were 110 and 0.3, respectively. These values indicate that the Xpert MTB/RIF test is reliable in identifying or ruling in pericarditis TB, and is a faster and easier method compared to other tests.

The strengths of our study were the use of standard protocols from the PRISMA guidelines and the Cochrane Handbook, strict inclusion and exclusion criteria, extraction of data by independent reviewers, and the use of bivariate models to calculate pooled sensitivity and specificity. However, we realized that our study has several limitations. We acknowledge that we might excluded numerous studies of extrapulmonary TB that did not have enough data on TB pericarditis despite comprehensive exploration. Furthermore, this meta-analysis involved a relatively low number of patients (581 eligible patients) due to the limited study of the use of Xpert MTB/RIF to diagnose pericarditis TB. Thus, the result might not reveal the actual state in the population. Further meta-analysis should be conducted when more studies specifically evaluate the use of Xpert MTB/RIF to diagnose pericarditis TB with a larger sample size.

## Data availability

### Underlying data

All data underlying the results are available as part of the article and no additional source data are required.

### Extended data

Figshare: Detailed search strategy. https://doi.org/10.6084/m9.figshare.11974356.v1 ^29^

Figshare: Review protocol. https://doi.org/10.6084/m9.figshare.11974260 ^30^

### Reporting guidelines

Figshare: PRISMA checklist for Diagnostic Test Accuracy of Xpert MTB/RIF for Tuberculous Pericarditis: A Systematic Review and Meta-Analysis https://doi.org/10.6084/m9.figshare.11955003.v1 ^31^

Data are available under the terms of the Creative Commons Attribution 4.0 International license (CC-BY 4.0).
