# Supplementary material for: Diagnostic test accuracy of Xpert MTB/RIF for tuberculous pericarditis: a systematic review and meta-analysis
Source: F1000Res. 2020 Jul 22;9:761. [Version 1] doi: 10.12688/f1000research.22770.1 (PMC7417956; doi:10.12688/f1000research.22770.1)
Supplement: Peer review history

# Peer review: Reviewer response for version 1

**DOI:** 10.5256/f1000research.25141.r67746

**Published:** 2020-08-10

**Authors:** Mohammad Saifur Rohman

**Affiliations:** 1 Departement of Cardiology and Vascular Medicine, Faculty of Medicine, Saiful Anwar General Hospital, Brawijaya University, Malang, Indonesia

The study is well written with objective and reliable method was used. It is acceptable for scientific standard. It is interesting looking at the result of this study. Which tried to do metanalysis from 9 research in diagnosing tuberculosis related pericardial effusion using Xpert MTB/RIF.

 The author also already considered bias risk and applicability concern.

Are the rationale for, and objectives of, the Systematic Review clearly stated?

Yes

Is the statistical analysis and its interpretation appropriate?

Yes

Are sufficient details of the methods and analysis provided to allow replication by others?

Yes

Are the conclusions drawn adequately supported by the results presented in the review?

Yes

Reviewer Expertise:

Cardiology Translational research : Pharmacogenetic, Interventional  cardiology

I confirm that I have read this submission and believe that I have an appropriate level of expertise to confirm that it is of an acceptable scientific standard.

# Peer review: Reviewer response for version 1

**DOI:** 10.5256/f1000research.25141.r67749

**Published:** 2020-08-06

**Authors:** Pieter A. Doevendans

**Affiliations:** 1 Divisio n of Hear t and Lun gs, Department of Cardiology, Uni versit y Medical Center Utrecht, Nether lands Heart Institute, University of Utrecht, Utrecht, The Netherlands

- The study is combing the available data on a diagnostic test to detect TB as a cause in this case for pericarditis. From the various studies it becomes clear that the specificity is excellent. Sensitivity is however disappointing, maybe due to a low bacterial load. So there is a place for the test in making a fast diagnosis to start early treatment. Yet a negative result does not exclude TB as the possible aetiology.
- Study is well designed well performed and not overstating the technique can be used for rule in. Well done work, well written.
- Only 1 typo on page 8: 
*we might excluded *
  No other corrections are needed at this point in time

Are the rationale for, and objectives of, the Systematic Review clearly stated?

Yes

Is the statistical analysis and its interpretation appropriate?

I cannot comment. A qualified statistician is required.

Are sufficient details of the methods and analysis provided to allow replication by others?

Yes

Are the conclusions drawn adequately supported by the results presented in the review?

Yes

Reviewer Expertise:

General cardiology

I confirm that I have read this submission and believe that I have an appropriate level of expertise to confirm that it is of an acceptable scientific standard.
